# Nanostructuring Biobased Epoxy Resin with PEO-PPO-PEO Block Copolymer

**DOI:** 10.3390/polym15051216

**Published:** 2023-02-28

**Authors:** Irati Barandiaran, Joseba Gomez-Hermoso-de-Mendoza, Junkal Gutierrez, Agnieszka Tercjak, Galder Kortaberria

**Affiliations:** 1 Group ‘Materials + Technologies’ (GMT), Chemical and Environmental Engineering Department, Faculty of Engineering of Gipuzkoa, University of the Basque Country (UPV/EHU), Plaza Europa 1, 20018 Donostia-San Sebastian, Spain; 2 Chemical and Environmental Engineering Department, Faculty of Pharmacy, University of the Basque Country (UPV/EHU), Paseo de la Universidad, 7, 01006 Vitoria-Gasteiz, Spain

**Keywords:** biobased, epoxy, block copolymer, nanostructuring

## Abstract

A biobased diglycidyl ether of vanillin (DGEVA) epoxy resin was nanostructured by poly(ethylene oxide-b-propylene oxide-b-ethylene oxide) (PEO-PPO-PEO) triblock copolymer. Due to the miscibility/immiscibility properties of the triblock copolymer in DGEVA resin, different morphologies were obtained depending on the triblock copolymer amount. A hexagonally packed cylinder morphology was kept until reaching 30 wt% of PEO-PPO-PEO content, while a more complex three-phase morphology was obtained for 50 wt%, in which large worm-like PPO domains appear surrounded by two different phases, one of them rich in PEO and another phase rich in cured DGEVA. UV-vis measurements show that the transmittance is reduced with the increase in triblock copolymer content, especially at 50 wt%, probably due to the presence of PEO crystals detected by calorimetry.

## 1. Introduction

During recent years, bio-based polymers have attracted attention due to the overuse of fossil fuels as well as the increase in greenhouse gas emissions, which causes important environmental issues [1,2]. Those polymers can be obtained from renewable materials, such as lignin [3] or vegetable oils [4,5], among others. Between these different types of polymeric materials, epoxy-based thermosets are the most popular, due to their broad spectrum of properties through the selection of epoxy prepolymers and curing agents, and therefore their use in various applications, such as coatings, adhesives, and composites, among others [6,7,8]. Over 90% of these epoxide materials are based on bis(4-hydroxyphenylene)-2,2-propane, known as bisphenol A, to which the aromatic ring confers good thermal resistance. Commercialized for more than 50 years, these bisphenol A based thermosets (DGEBA) have been employed in many common products, such as containers, and human health applications, such as filling materials or sealants in dentistry. However, bisphenol A can also mimic the body’s own hormones, and it could lead to severe negative health effects [9,10], besides cited environmental issues. Recently, poly-functional glycidyl ether derivatives based on both biobased and barely toxic extracts, such as vanillin [11,12,13,14,15] and phloroglucinol [16,17,18], which are extracted from lignins and tannins [1,19], and used as food flavoring or active ingredient in medicine, have been studied as new feedstock for thermosets. Between different biobased resins investigated by other authors, diglycidyl ether of vanillin (DGEVA) have shown good thermomechanical properties [20,21].

On the other hand, the self-assembly of block copolymers (BCP) into different nanoscale structures makes them interesting polymeric macromolecules from both academic and industrial points of view. This class of macromolecules consists of two or more covalently linked polymers, which are thermodynamically incompatible, giving rise to a variety of the microstructures. As it is well known, BCPs can self-assemble to form nanoscale structures with domain spacing that depends strongly on molecular weight, segment size and interaction between the blocks among others. Consequently, microphase separation of BCPs is determined by the degree of polymerization, *N*, the volume fraction of each block, *f*, and the Flory–Huggins interaction parameter, χ, which depends on temperature. A typical size of the microphase separated BCP domains is in the range of 10–200 nm.

BCPs can microphase separated in stable structures, such as lamellar, hexagonal-packed cylinder, body-centered cubic, close-packed spherical, or bicontinuous cubic gyroid structures. The ability to control both the length scale and the spatial organization of BCP morphologies makes these polymeric materials attractive candidates for use as templates for the fabrication of novel multifunctional materials with application in many fields of nanotechnology and advanced materials.

BCPs can also act as a nanostructuring agent for different homopolymers and thermosetting systems. As the main drawback of epoxy thermosetting polymers, for their applications as adhesives, surface coatings or composite matrices, is their low fracture toughness. One of the successful pathways to achieve high improvements on the toughness of these systems is incorporation of BCPs [22,23,24,25,26]. Use of the BCPs not only improves the toughness of thermosetting polymers but also leads to nanostructured thermosets, which can act as templates for dispersion and selective localization of low molecular weight organic molecules, such as azobenzene or liquid crystals, or inorganic nanoobjects, such as nanoparticles, carbon nanotubes, nanofibers and others.

Nanostructured thermosetting materials can be formed followed two different mechanisms. In the first one, the epoxy precursor acts as a selective solvent and, consequently, the microphase separation takes place before the curing reaction, and the epoxy network formation process only fixed the final morphology. In the second pathway, the microphase separation of the immiscible block takes place by reaction-induced phase separation (RIPS). Thus, the mixture of BCP and epoxy precursors is miscible before curing and the phase separation takes place during network formation.

Many authors, among which our research group can be mentioned, obtained nanostructured thermosetting systems by employing amphiphilic BCPs. Different amphiphilic BCPs used as nanostructuring agents by different authors can be found in Table 1.

As it can be seen in Table 1, poly(ethylene glycol)-poly(propylene glycol)-poly(ethyleneglycol) (PEO-PPO-PEO), has been widely employed for nanostructuring epoxy matrices, mainly DGEBA resin [30,31,32,33,34,35]. The popularity of PEO-PPO-PEO is due to its commercial availability, including different ratios of each block as well as the simplicity of the experimental procedure and the absence of any chemical synthesis or reaction with the epoxy system [32]. For PEO-PPO-PEO/epoxy blends, the formation of the self-assembled nanostructure depends on the curing conditions, curing agent and the inner characteristics of the BCP [53]. Regarding the effect of BCP composition, Guo et al. [30] obtained different nanostructured features based on the DGEBA/MDA system and PPO-PEO-PPO copolymers with different block ratios. For the BCP with 30 wt% of PEO block, it did not find macroscopic phase separation up to a content of 50 wt%, exhibiting nanostructures based on spherical PPO domains with an average size of about 10 nm. For blends with the BCP with 80 wt% PEO, blends were not macroscopically phase-separated over the entire composition range because of the much higher PEO content, showing composition-dependent nanostructures on the order of 10−100 nm. Sun et al. [31] studied the same systems by solid-state nuclear magnetic resonance (NMR), finding that the domain size and long period depended strongly on the PEO fraction. They demonstrated that PEO blocks were only partially miscible with the cured network. Upon curing, the cross-linked rigid epoxy resin formed a separated microphase, while some PEO were locally expelled out of the cured network, forming another microphase with PPO. Similar systems but employing diamino diphenyl methane (DDM) as a hardener have also been deeply analyzed by our group [32,33,34,35]. Firstly, the miscibility and morphological features were studied, together with cure kinetics, by changing cure temperatures and copolymer amount [32]. Depending on the curing condition, phase separation took place at micro or nanoscale due to competition among kinetic and thermodynamic factors. Two distinct phases were present for every blend studied except for the system with 10 wt% PEO–PPO–PEO and cured at a low temperature. A thermodynamic model describing a thermoset/block copolymer considered as only one entity system was proposed. In a second stage, the effect of copolymer composition (block ratios) and curing conditions was analyzed [33]. A delay of cure rate was found, which increased as copolymer content and PEO molar ratio in the block copolymer increased. Infrared spectroscopy showed that PEO block was mainly responsible for physical interactions between the hydroxyl groups of growing epoxy thermoset and ether bonds of block copolymer that led to the delay in cure kinetics. Regarding structural characterization [34], taking into account DGEBA/DDM systems modifided with PEO or PPO homopolymers for comparison, it was found that, depending on the molar ratio among blocks, micro or macrophase separated morphologies were obtained. For high molar ratio among blocks, microphase-separated structures were obtained for all block copolymer contents and cure temperatures, with the self-assembly of PPO into nanoscopic entities. For low molar ratio among blocks, however, the physical interactions among the PEO block and the epoxy matrix were not favourable enough, due to the lower content of this block. Indeed, the micelles formed initially coalesced, leading to macroscopic phase separation, where different morphologies were obtained depending on copolymer content and cure temperature. Finally, the mechanical properties–morphology relationships were also analyzed [35]. Macrophase-separated systems modified with low PEO/PPO block ratio showed a similar behaviour to that for rubber-modified systems. Increasing the content of a modifier decreases both flexural modulus and strength, while fracture toughness increases. Microphase-separated systems, on the other hand, did not present significant changes in both flexural modulus and strength for low contents, but the critical stress intensity factor increased due to partial miscibility of the PEO block with the epoxy matrix.

Parameswaranpillai et al. [54] nanostructured a DGEBA/DDM system with PEO-PPO-PEO, finding that the phase separation occurred via self-assembly of PPO blocks, followed by the reaction-induced phase separation of PEO blocks, and confirming that phase separated PEO blocks formed the crystalline phase in the amorphous crosslinked epoxy matrix.

In the present work, as a preliminary study for analyzing the nanostructuring of bio-based epoxy thermosetting formulation, DGEVA epoxy resin has been modified using different amounts of PEO-PPO-PEO triblock copolymer ranging from 10 to 50 wt%. Thermal properties are analyzed in terms of differential scanning calorimetry (DSC) and thermogravimetric analysis (TGA), while optical properties are characterized by UV-vis spectroscopy and corroborated by photographs. Finally, the effect of copolymer amount on the morphology of the nanostructured thermosetting system is analyzed by atomic force microscopy (AFM). The possibility of nanostructuring and the control of generated nanostructures will be further employed in future works for the preparation of ternary systems based on biobased epoxy thermosetts by placing nanofillers at the nanodomains.

## 2. Materials and Methods

### 2.1. Materials and Sample Preparation

The biobased epoxy used in this research work was diglycidyl ether of vanillin (DGEVA) supplied by Specific Polymers, Castries, France. The curing agent was 4,4′-diaminodiphenylmethane (DDM), purchased from Sigma-Aldrich, Darmstadt, Germany. The block copolymer used was poly(ethylene oxide-b-propylene oxide-b-ethylene oxide) (PEO-PPO-PEO) triblock copolymer (Pluronic F-127) supplied by Sigma Aldrich, Darmstadt, Germany. Chemical structures of employed materials are shown in Table 2. An amine:epoxy ratio of 1:1 was used for the DGEVA/DDM system, while PEO-PPO-PEO block copolymer content was varied from 10 to 50 wt% to design different BCP-DGEVA/DDM systems.

Sample preparation was carried out in the following way. First, DGEVA resin and PEO–PPO–PEO triblock copolymer were blended at 80 °C under mechanic stirring. Then, a corresponding amount of DDM was added with continuous stirring, in an oil bath at 80 °C, until a homogeneous mixture was achieved. Finally, the mixture was poured to the mold, and samples were degassed in a vacuum oven and cured at 120 °C for 6 h and post-cured under vacuum at 190 °C for 2 h. In both cases, a mechanical vacuum pump device was employed.

### 2.2. Techniques

Differential scanning calorimetry (DSC) measurements of the individual components, as well as BCP-DGEVA/DDM systems, were performed using a DSC3+ from Mettler Toledo equipment (Columbus, OH, USA). Thermal behavior of individual components and the DGVA/DDM system was evaluated by dynamic scans performed from −80 °C to 250 °C at 10 °C/min scan rate. The miscibility of PEO–PPO–PEO triblock copolymer with the uncured DGEVA/DDM system was investigated by dynamic scans performed from −80 °C to 50 °C at 10 °C/min scan rate. The curing processes of all BCP-DGEVA/DDM systems were analyzed by isothermal scan performed at 80, 100 and 120 °C (followed by a dynamic scan from 25 °C to 200 °C at 10 °C/min). Finally, thermal behavior of BCP-DGEVA/DDM systems was analyzed by dynamic scans performed from −80 °C to 250 °C at 10 °C/min scan rate. All experiments were performed under nitrogen atmosphere, with a flow of 10 mL/min.

Thermogravimetric tests were performed on a TGA 500 from TA Instruments Inc. (New Castle, DE, USA). Samples were heated from 25 to 800 °C at a heating rate of 10 °C/min under nitrogen atmosphere.

Fourier-transformed infrared spectroscopy (FTIR) spectra were recorded with a Nicolet Nexus spectrometer from Thermo Fisher Scientific SL (Bilbao, Spain) with a Golden Gate ATR sampling accessory. Background was recorded before every sample and the spectra were obtained in the range of 4000–650 cm^−1^, performing 32 scans with a resolution of 4 cm^−1^.

The morphologies of the cured BCP-DGEVA/DDM systems were studied by atomic force microscopy (AFM) under ambient conditions, using a scanning probe microscope Multimode 8 from Bruker (Billerica, MI, USA). Tapping mode (TM) was employed in air using an integrated tip/cantilever (125 mm in length with a 300 kHz resonant frequency). Measurements were performed with 512 scan lines and target amplitude around 0.9 V. Different regions of the cured BCP-DGEVA/DDM systems were scanned to ensure that the morphology of the investigated materials was a representative one. Samples were cut using an ultramicrotome Leica Ultracut R with a diamond blade.

UV-vis transmittance spectra of the cured BCP-DGEVA/DDM systems were performed with a Shimadzu UV-3600 (Kioto, Japan) spectrophotometer in the range between 200 and 800 nm.

## 3. Results and Discussion

### 3.1. Differential Scaning Calorimetry Analysis

DSC dynamic measurements were carried out in order to investigate the miscibility between PEO-PPO-PEO triblock copolymer and DGEVA/DDM.

**Figure 1:**
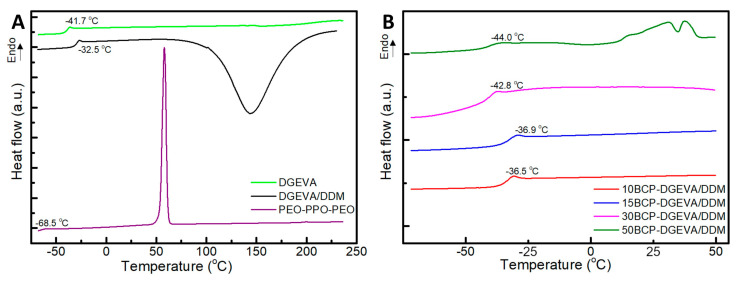
Dynamic DSC thermograms of (**A**) the neat components (DGEVA resin and PEO-PPO-PEO triblock copolymer) and uncured DGEVA/DDM blend, and (**B**) uncured BCP-DGEVA/DDM blends with different BCP contents from 10 to 50 wt%. Note: thermograms have been shifted along the Y-axes for a better visualization.

Figure 1A shows thermograms of blend components (PEO-PPO-PEO and DGEVA/DDM). Moreover, uncured BCP-DGEVA/DDM blends with different BCP amounts were also included in Figure 1B. PEO-PPO-PEO thermogram shows a T_g_ at around −68.5 °C [32] and a melting peak centered at 58.0 °C, related to the melting of crystalline PEO block. DGEVA resin presents a T_g_ at −41.7 °C that increased up to −32.5 °C when curing agent was added. This behavior can be related to the partial miscibility between the DGEVA resin and the amine before curing. In addition, the DGEVA/DDM thermogram shows an exothermic peak centered around 140 °C, indicating the curing reaction of the blend. If the dynamic thermograms for BCP-DGEVA/DDM systems are compared, the T_g_ of the DGEVA resin phase decreases from −36.5 °C to −44.0 °C with increasing BCP content, owing to the miscibility of PEO-PPO-PEO and DGEVA [32,37]. Moreover, at the thermogram of the 50BCP-DGEVA/DDM system, the melting of the crystalline phase of the PEO block is detected and, in contrast to that of neat BCP, the melting happens in two steps, indicating the presence of different types of crystals. This phenomenon will be further discussed in the morphology section shown below.

**Figure 2:**
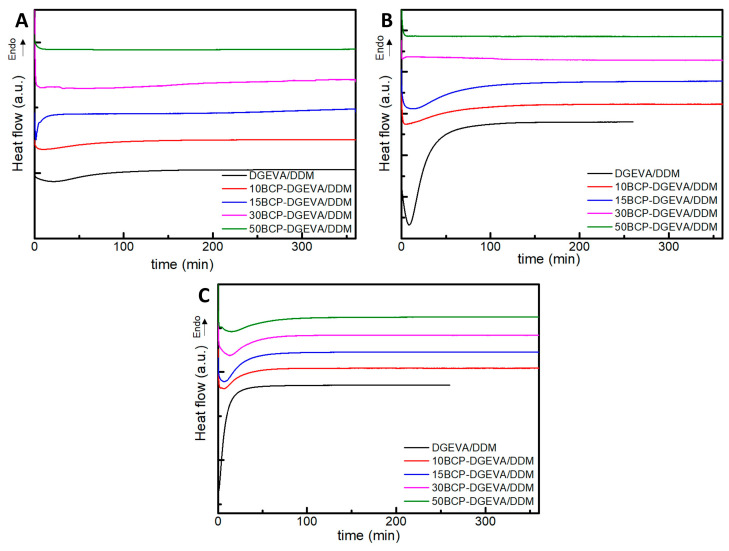
Isothermal DSC thermograms of investigated BCP-DGEVA/DDM systems with BCP content from 0 to 50 wt% at (**A**) 80, (**B**) 100, and (**C**) 120 °C. Note: thermograms have been shifted along the Y-axes for a better visualization.

The curing behavior of all BCP-DGEVA/DDM blends was analyzed by isothermal thermograms at 80, 100 and 120 °C (Figure 2).

As can be observed, the reaction rate increased with the increasing of curing temperature, while BCP addition delayed the exothermic peak of the isothermal thermograms at all temperatures, due to the dilution effect of PEO-PPO-PEO [32,46]. For systems with highest amount of BCP, the exothermic peak almost disappeared at 80 and 100 °C, as the full conversion was not reached in the analyzed time scale at 80 and 100 °C. For these BCP-DGEVA/DDM systems, the curing process would be completed at the post-curing stage. At 120 °C the curing reaction was completed for all investigated BCP-DGEVA/DDM systems, and all composites were cured at 120 °C.

**Figure 3:**
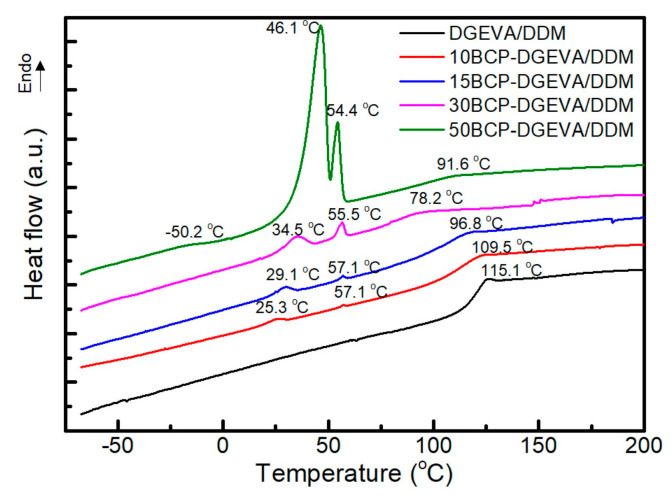
Dynamic DSC thermograms of investigated BCP-DGEVA/DDM systems with BCP content from 0 to 50 wt% cured at 120 °C. Note: thermograms have been shifted along the Y-axes for a better visualization.

All BCP-DGEVA/DDM systems cured at 120 °C were studied by dynamic DSC analysis (Figure 3). The neat DGEVA/DDM epoxy system showed a T_g_ at 115.1 °C. With PEO-PPO-PEO triblock copolymer addition, the T_g_ of the epoxy matrix decreased from 109.5 °C (10BCP-DGEVA/DDM system) to 91.6 °C (50BCP-DGEVA/DDM system), confirming of the epoxy matrix the miscibility between DGEVA epoxy resin and PEO block of PEO-PPO-PEO [26]. However, the sample with 30 wt% of BCP presented a lower T_g_ value of for epoxy matrix than the sample with 50 wt% of BCP. Moreover, the 50BCP-DGEVA/DDM system presented an additional T_g_ at −50.2 °C, which could be attributed to the T_g_ of PEO block of PEO-PPO-PEO. Dynamic thermograms of BCP-DGEVA/DDM systems with BCP content from 10 to 50 wt% also presented endothermic peaks, related to the melting of PEO block of the BCP, which is represented by two peaks that tend to become closer as the BCP content is increased, indicating different types of crystals.

As has been pointed out by other authors [26,33], the T_g_ of a blend depends on the weight fraction of the components. For this reason, it could be expected that the higher the BCP content in the mixture, the lower the T_g_ of the system will be. However, in this case, although the BCP amount is higher for the 50BCP-DGEVA/DDM system than for the 30BCP-DGEVA/DDM one, the value of the T_g_ increases. This could indicate that part of the PEO block phase separates from the DGEVA/DDM matrix, as found in previous works of our group [26]. These melting peaks increased significantly in the case of the 50BCP-DGEVA/DDM system. The increase in melting peaks, together with the presence of the T_g_ of PEO block of BCP and the higher T_g_ of epoxy matrix when compared with that for the 30BCP-DGEVA/DDM system, could indicate that the phase separated BCP content could be remarkably higher in this case than for the rest of the systems.

### 3.2. Thermogravimetric Analysis

**Figure 4:**
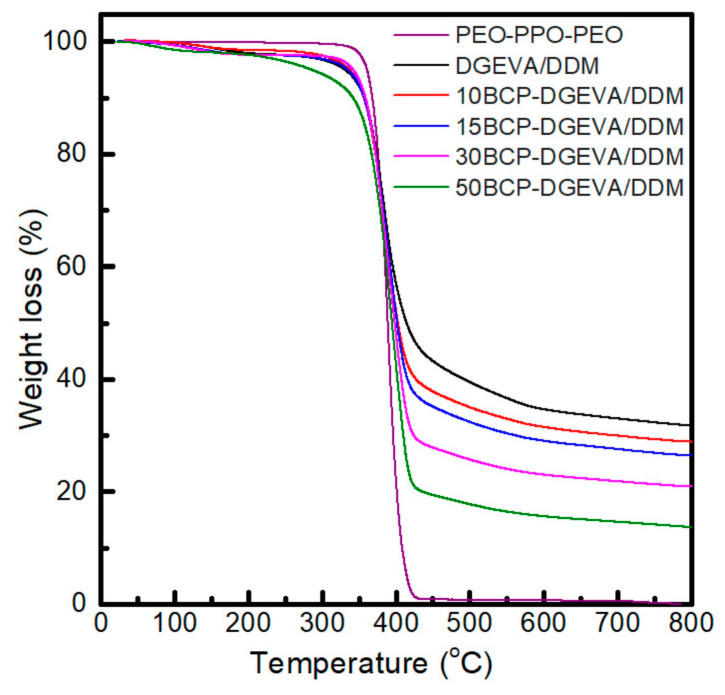
TGA curves of PEO-PPO-PEO block copolymer and BCP-DGEVA/DDM systems with BCP content from 0 to 50 wt% cured at 120 °C.

Thermogravimetric analysis of PEO-PPO-PEO triblock copolymer and developed BCP-DGEVA/DDM systems was also carried out (Figure 4). If the thermal degradation curves of PEO-PPO-PEO and neat DGEVA/DDM are compared, for both samples the main degradation step occurs between 350 and 425 °C. As a result, for BCP-DGEVA/DDM systems the main degradation occurs in the same temperature range, showing that BCP addition does not affect the thermal stability of the system. On the other side, formed char amount (32 wt% for neat epoxy system) proportionally decreases with BCP content from 29 wt% for the 10BCP-DGEVA/DDM system to 14 wt% for the 50BCP-DGEVA/DDM one.

### 3.3. Fourier-Transform Infrared Spectroscopy Analysis

**Figure 5:**
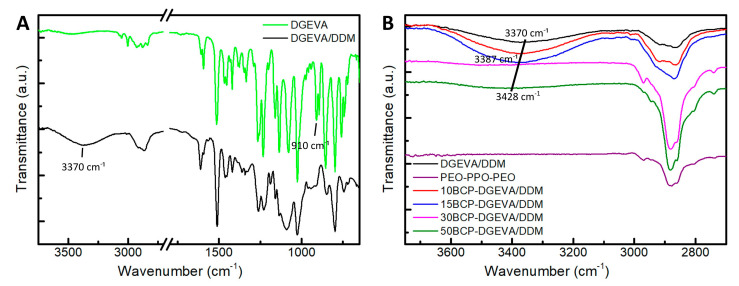
FTIR spectra of (**A**) neat DGEVA resin and DGEVA/DDM system, and (**B**) investigated BCP-DGEVA/DDM systems with BCP content from 0 to 50 wt% cured at 120 °C. Note: spectra have been shifted along the Y-axes for a better visualization.

Figure 5A shows FTIR spectra of DGEVA resin and the DGEVA/DDM system. If these two spectra are compared, in the case of DGEVA/DDM system, a broad band centered at 3370 cm^−1^ is detected, attributed to the alcohol groups formed after the reaction of the epoxy groups of DGEVA and the amine groups of DDM [33]. In addition, the peak related to the epoxide group (910 cm^−1^) disappears at the cured spectrum, proving the curing of the epoxy resin [33,55].

Regarding the effect of PEO-PPO-PEO triblock copolymer addition, Figure 5B shows that by increasing BCP content, the intensity of the band related to alcohol groups (broad band centered at 3370 cm^−1^, in DGEVA/DDM) decreases and shifts towards higher wavenumbers (3387 and 3428 cm^−1^ for 15BCP-DGEVA/DDM and 50BCP-DGEVA/DDM, respectively). This could be due to the hydrogen bonding interaction between the OH groups formed in the cured resin and the PEO block of the triblock copolymer [33]. Moreover, in the spectra of systems with higher BCP content (30 and 50 wt%) the bands related to the PEO-PPO-PEO block copolymer present higher intensity.

### 3.4. Atomic Force Microscopy

**Figure 6:**
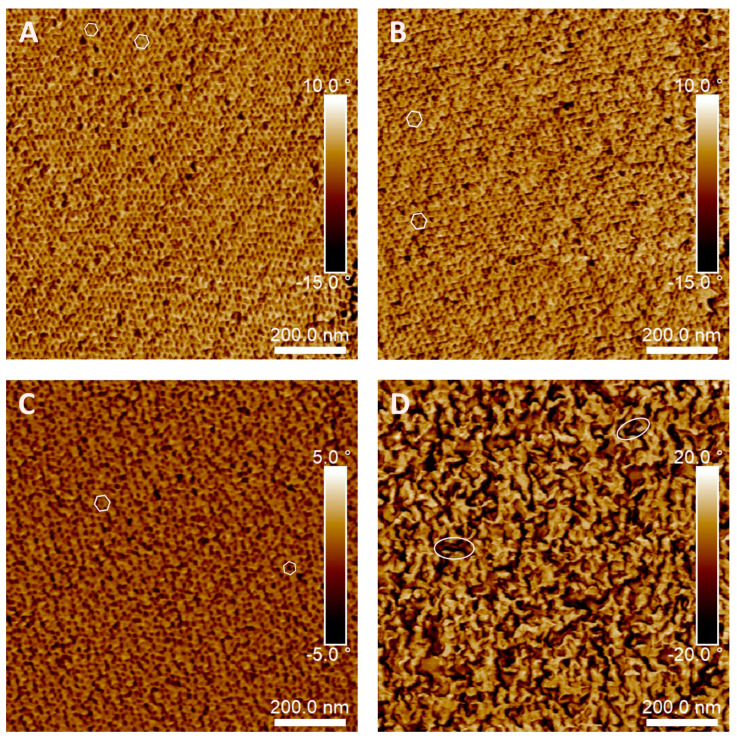
AFM phase images of BCP-DGEVA/DDMP systems cured at 120 °C with (**A**) 10 wt%, (**B**) 15 wt%, (**C**) 30 wt%, and (**D**) 50 wt% of PEO-PPO-PEO.

The morphologies of the BCP-DGEVA/DDM systems cured at 120 °C were investigated by AFM. As can be observed in Figure 6, all investigated systems show microphase separation at nanoscale. In the case of the samples with BCP content up to 30 wt%, it is remarkable the formation of small nanostructures. For the system with 10 wt% of block copolymer (Figure 6A), a hexagonally packed cylinder morphology is formed, in which the dark spherical domains with diameters ranging from 10 to 15 nm correspond to the PPO block rich phase, while the continuous light phase corresponds to the PEO-epoxy rich one [31]. As other authors have reported for DGEBA epoxy systems [26,37,46], it seems that as a result of the interactions between the PEO block and epoxy resin, the PEO block is miscible with DGEBA epoxy resin, while PPO remains immiscible. In the case of the system based on DGEVA resin, a similar behavior is observed. As shown in Figure 6B, an increase of 5 wt% in PEO-PPO-PEO triblock copolymer content seems not to affect the morphology observed, as the 15BCP-DGEVA/DDM system presents the same morphology than the 10BCP-DGEVA/DDM one. Moreover, for the 30BCP-DGEVA/DDM system, no significant morphological changes are detected, observing a similar hexagonally packed cylinder morphology (marked at the images) than for 10BCP-DGEVA/DDM and 15BCP-DGEVA/DDM systems. On the contrary, when PEO-PPO-PEO concentration rises up to 50 wt%, the morphology changes drastically. In this case, large worm-like domains (PPO block) are observed, surrounded by two different phases, one of them rich in PEO (lower hardness) and the last phase rich in cured DGEVA [56]. This fact could be in agreement with the dynamic DSC results for the 50BCP-DGEVA/DDM system shown in Figure 3, in which an additional T_g_ attributed to the PEO block of PEO-PPO-PEO was detected.

### 3.5. UV-Vis Spectroscopy

**Figure 7:**
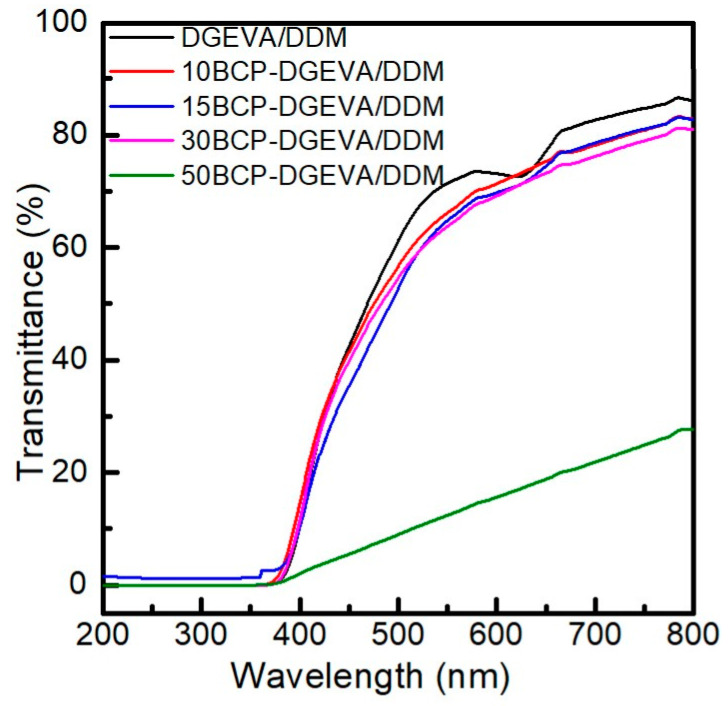
UV-vis transmittance results of BCP-DGEVA/DDM systems with BCP content from 0 to 50 wt% cured at 120 °C.

UV-vis transmittance results of the BCP-DGEVA/DDM systems with different PEO-PPO-PEO contents are shown in Figure 7. The transmittance of the DGEVA/DDM system decreases with the addition of triblock copolymer. The DGEVA/DDM system presents a transmittance value of 77% at 650 nm, while values of 75, 74 and 73% are measured for systems with 10, 15 and 30% of BCP, respectively. When the triblock copolymer content increases to 50 wt%, the transmittance value at 650 nm is reduced to 19%. The presence of PEO crystals observed by DSC for this system (increase in the melting temperature for PEO block) could explain the drastic reduction in the transmittance.

**Figure 8:**
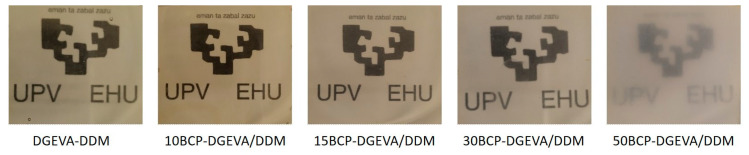
Photographs of all BCP-DGEVA/DDM samples cured at 120 °C.

The digital images of samples shown in Figure 8 corroborate the results obtained by UV-vis transmittance. The systems with PEO-PPO-PEO concentrations up to 30 wt% allow light transmittance, while the 50BCP-DGEVA/DDM system presents much lower transmittance and the image behind cannot be clearly seen.

## 4. Conclusions

The following conclusions can be extracted from this preliminary work on the nanostructuring of bio-based epoxy matrix by PEO-PPO-PEO block copolymer.

This research work demonstrates that the biobased DGEVA epoxy resin is an adequate resin to be nanostructured with PEO-PPO-PEO triblock copolymer. The curing temperature was set at 120 °C, as at lower temperatures systems with higher BCP content did not reach full conversion, as BCP addition delayed the cure reaction by dilution effect. The investigated systems showed, up to 30 wt% of triblock copolymer, a hexagonally packed cylinder morphology, with spherical domains ranging from 10 to 15 nm. In the 50BCP-DGEVA/DDM system, a change in the morphology was detected, forming a more complex morphology with phase separation of PEO-PPO-PEO triblock copolymer. These results are in agreement with presented DSC thermograms, in which an additional T_g_ related to PEO crystals was detected, and also with the transmittance data obtained by UV-vis, as the most significant decrease in transmittance was not detected up to a BCP content of 50 wt%, probably due to the presence of PEO crystals.

## Figures and Tables

**Table 1:** Relation of different amphiphilic block copolymers and thermosetting precursors used by different authors.

BCPs	Abbreviation	Thermosetting Precursor	References
poly(hexylene oxide)-b-poly(ethylene oxide)	PHO-b-PEO	DGEBA + PN	[24]
poly(ethylene oxide)-b-poly(ethyl ethylene)	PEO-b-PEE	DGEBA + PA	[27]
poly(ethylene oxide)-b-poly(ethylene-alt-propylene)	PEO-b-PEP	DGEBA + MDA	[28]
poly(ethylene oxide)-b-poly(propylene oxide)	PEO-b-PPO	DGEBA + MDA	[29]
poly(ethylene oxide)-b-poly(propylene oxide)-b- poly(ethylene oxide)	PEO-b-PPO-b-PEO	DGEBA + MDA DGEBA + DDM	[30,31] [32,33,34,35]
polyethylene-b-poly(ethylene oxide)	PE-b-PEO	DGEBA + MDA DGEBA + MCDEA	[36] [37]
poly(ethylene oxide)-b-poly(dimethylsiloxane)	PEO-b-PDMS	DGEBA + MDA	[38]
poly(ethylene oxide)-b-poly(ε-caprolactone)	PEO-b-PCL	DGEBA + MOCA	[39]
poly(ethylene oxide)-b-polystyrene	PEO-b-PS	DGEBA + MDA DGEBA + MXDA DGEBA + MCDEA DGEBA + DDM	[40] [41,42,43,44] [45,46] [47]
poly(*ε*-caprolactone)-b-polybutadiene-b-poly(*ε*-caprolactone)	PCL-b-PB-b-PCL	DGEBA + MOCA	[48]
poly(ε-caprolactone)-b-poly(n-butyl acrylate)	PCL-b-PBA	DGEBA + MOCA	[49]
poly(heptadecafluorodecyl acrylate)-b-poly(caprolactone)	PaF-b-PCL	DGEBA + MCDEA	[50]
polydimethylsiloxane-b-poly(ε-caprolactone)-b-polystyrene	PDMS-b-PCL-b-PS	DGEBA + MOCA	[51]
poly(ε-caprolactone)-b-polystyrene	PCL-b-PS	DGEBA + MOCA	[52]

**Table 2:** Chemical structures of employed materials.

Material	Chemical Structure
DGEVA	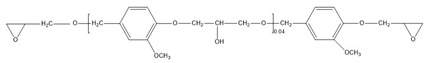
DDM	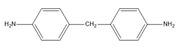
PEO-PPO-PEO	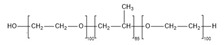

## Data Availability

The data presented are available on request from the corresponding author.
